# The prognostic value of preoperative plasma fibrinogen in Asian patients with urothelial cancer: a systematic review and meta-analysis

**DOI:** 10.3389/fendo.2024.1360595

**Published:** 2024-08-29

**Authors:** Zhengqing Bao, Guizhong Li, Feng He, Xiao Xu, Zhenhua Liu, Jianwei Wang

**Affiliations:** Department of Urology Surgery, Beijing Jishuitan Hospital, Capital Medical University, Beijing, China

**Keywords:** plasma fibrinogen, urothelial cancer, prognosis, meta-analysis, Asian

## Abstract

**Objective:**

We conducted this meta-analysis to comprehensively explore the prognostic value of the preoperative plasma fibrinogen in Asian patients diagnosed with urothelial cancer (UC).

**Methods:**

After a systematic search of Web of Science, PubMed, and Embase before May 2024, we included 10 studies in our meta-analysis. The hazard ratios (HRs) with 95% confidence interval (CI) for overall survival (OS), cancer-specific survival (CSS), recurrence-free survival (RFS), and progression free survival (PFS) were estimated using fixed effect model.

**Results:**

This meta-analysis included a total of 2875 patients. UC patients with an elevated preoperative plasma fibrinogen had worse OS (pooled HR: 2.13, 95% CI: 1.81-2.51; P<0.001), CSS (pooled HR: 2.22, 95% CI: 1.83-2.70; P<0.001), RFS (pooled HR: 1.90, 95% CI: 1.59-2.27; P<0.001), and PFS (pooled HR: 2.12, 95% CI: 1.36-3.29, P=0.001). No significant heterogeneity or publication bias was found. Additionally, statistically significant pooled HRs were also calculated in subgroup analysis when stratified by cancer type, country, and cut-off value.

**Conclusions:**

The presence of elevated preoperative plasma fibrinogen levels is significantly correlated with unfavorable tumor outcomes in UCs.

## 1 Introduction

Urothelial carcinoma (UC) is one of the most common malignancies arising from the entire urinary tract (1), and it mainly includes bladder cancer (BC) and upper tract UC (UTUC). In United States, approximately 168,560 individuals will be diagnosed with UC and 32,590 will die from the disease in 2023 (2). The biological behavior of UC is complicated, making it prone to invasion, recurrence, and metastasis (3). Despite significant improvements in diagnosis and treatment of UC, oncologic outcomes remain poor. The 5-year survival rates for locally advanced UC and metastatic UC were only 34% and 5.4%, respectively (3). Therefore, an effective and applicable biomarker is necessary to accurately predict the prognoses and formulate follow-up strategies based on the stratification of risks for UC patients.

The plasma fibrinogen, serving as a crucial factor in blood coagulation and an indicator of inflammation, plays a pivotal role in maintaining human health (4, 5). Numerous studies have revealed that the coagulation/fibrinolytic system is initiated *in vivo* among cancer patients, and these markers can be employed for predicting tumorigenesis and prognosis (6, 7). Recently, an increasing body of evidence suggests that preoperative plasma fibrinogen can be used as a prognostic predictor in patients with UC, including UTUC (8–13) and BC (14–17). Song et al. (18) conducted a meta-analysis in urological cancers to assess the prognostic value of preoperative plasma fibrinogen. However, their study solely focused on UTUC (no BC, another type of UC) and had limited inclusion of studies. Interestingly, there are well-documented race-based differences in the treatment and outcomes for UC (19). Thus, the present meta-analysis included additional recent studies on UTUC and BC patients to evaluate the prognostic value of preoperative plasma fibrinogen on survival outcomes in UCs among Asian population.

## 2 Materials and methods

### 2.1 Protocol

Before commencing our study, we registered our systematic review project with the International Prospective Register of Systemic Reviews (PROSPERO; http://www.crd.york.ac.uk/PROSPERO/CRD42024496302). This meta-analysis was conducted in accordance with the Meta-analysis of Observational Studies in Epidemiology (MOOSE) criteria (see **Supplementary Files**).

### 2.2 Literature search

We systematically searched Web of Science, Embase, and PubMed to obtain all available clinical studies published before May 2024 without any language restrictions. We used the terms (fibrinogen, transitional cell carcinoma, upper urinary tract, urothelial carcinoma, ureter cancer, ureteral cancer, ureter carcinoma, ureteral carcinoma, bladder cancer, bladder carcinoma, and bladder tumor) to search for the related articles in the databases (see **Supplementary Files**). The literature search was independently conducted by two investigators, Zhengqing Bao and Guizhong Li.

### 2.3 Inclusion and exclusion criteria

The literature search, study selection, and validation were independently performed by two authors (Zhengqing Bao and Guizhong Li), and a third author (Jianwei Wang) was consulted to resolve the disagreements.

Studies were considered eligible if they met all of the following criteria: 1) cohort studies on patients with localized UCs reported the association between the preoperative plasma fibrinogen and oncological outcomes, included overall survival (OS), cancer-specific survival (CSS), recurrence-free survival (RFS), or/and progression free survival (PFS), after surgery. The plasma fibrinogen was tested before a definitive operation or diagnostic procedure (a definitive operation was generally performed shortly thereafter); 2) publications provided sufficient information to extract or calculate hazard ratios (HRs) and their corresponding 95% confidence intervals (CIs); 3) all patients classified into low and high plasma fibrinogen groups; and 4) full-text articles. The following studies were excluded based on any of the following criteria: 1) reviews, abstracts, letters, reviews, case reports, editorials, or basic studies; 2) studies with insufficient information for HRs and 95% CIs; 3) sample size<50; 4) non- Asian population; and 5) duplicate or overlapping studies.

### 2.4 Data extraction and quality assessment

The data extraction was independently performed by two investigators, Zhengqing Bao and Guizhong Li. The extracted data included the first author’s name, publication year, country, cancer type, sample size, duration time, age, gender, cutoff value, follow-up duration, HRs (95%CI), and analysis method (univariate/multivariate). If both univariate and multivariate analyses were performed, we chose the HRs (95%CI) from multivariate analysis. OS, CSS, RFS and PFS were analyzed. The study quality was systematically evaluated according to the Newcastle-Ottawa Scale (NOS) (see **Supplementary Files**). A ‘star system’ has been developed in which a study is judged on three broad perspectives: the selection of the study groups; the comparability of the groups; and the ascertainment of either the exposure or outcome of interest for studies respectively. A study can be awarded a maximum of one star for each numbered item within the Selection and Outcome categories. A maximum of two stars can be given for Comparability. Studies with more than 6 stars were considered as high-quality. The quality assessment was independently performed by two investigators (Zhengqing Bao and Guizhong Li), with a third reviewer (Jianwei Wang) consulted to resolve any disagreements.

### 2.5 Statistical analysis

All statistical analyses were conducted using STATA 15.0 (STATA Corporation, College Station, TX, USA). Statistical significance was set as a p-value<0.05. The heterogeneity of included studies was evaluated using Cochran’s Q-test and Higgins I^2^ statistics (I^2^). The random effects-model was used for the significant heterogeneity, which was indicated by I^2^>50% or P<0.05. Otherwise, the fixed-effect model was adopted to calculate pooled HRs for no obvious heterogeneity. Subgroup and meta-regression analyses were performed to explore the potential factors for heterogeneity. Sensitivity analysis was conducted using a “one-study removed” model to assess the stability of the overall results. Potential publication bias was assessed by using funnel plots visually, whose results were confirmed by using Begg’s and Egger’s tests. If significant publication bias was identified, the trim-and-fill method estimated an adjusted effect size.

## 3 Results

### 3.1 Characteristics of the included studies

**Figure 1:**
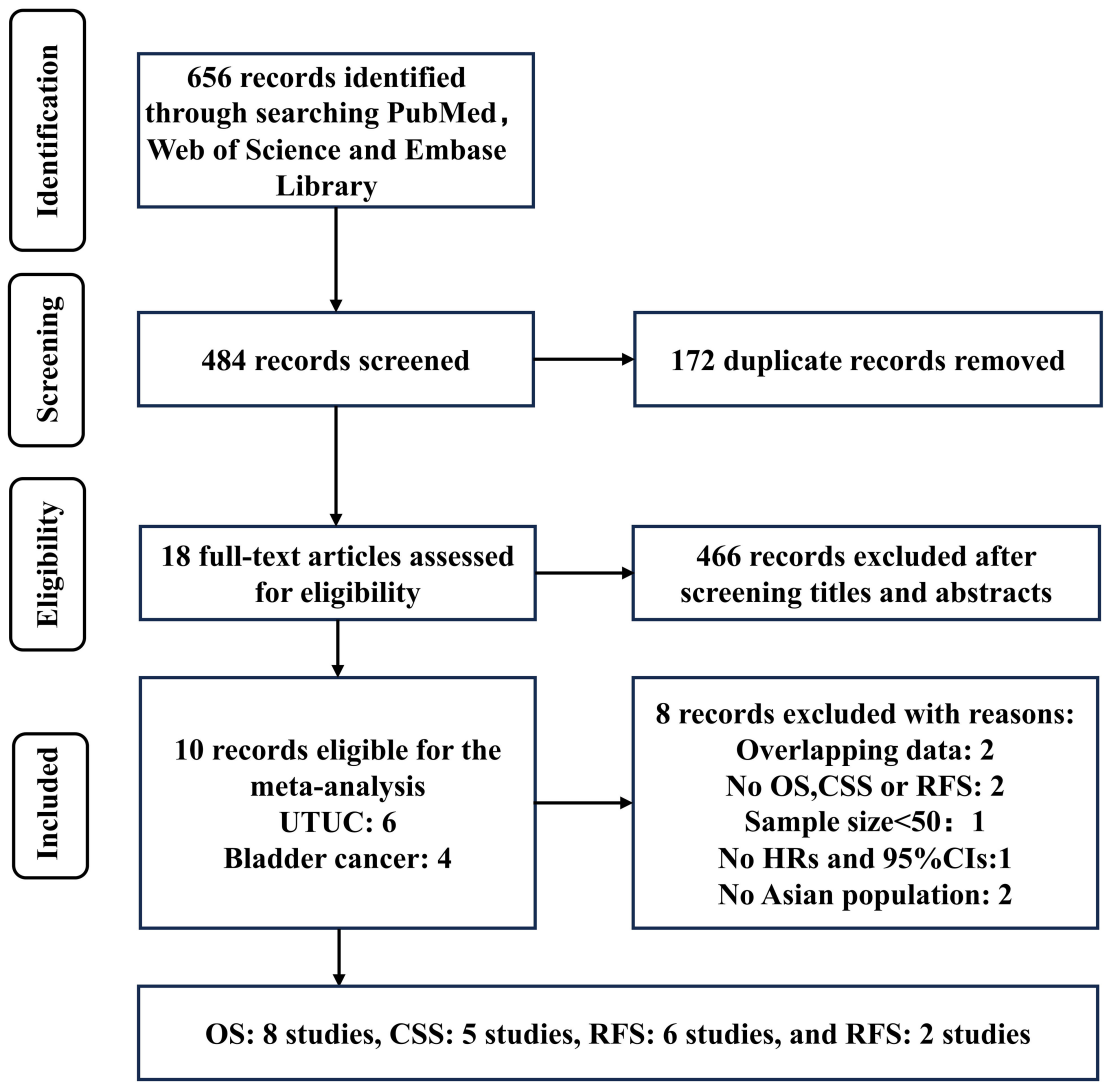
Flow diagram of the study selection process.

A total of 656 records were identified through a systematic literature search. After excluding 172 duplicates, the titles and abstracts of the remaining 466 records were screened, resulting in the selection of 18 articles for full-text reading. Out of these 18 records, 8 were excluded: two studies included overlapped data with others; two studies did not have OS, CSS, RFS, and PFS as final outcomes; one study had a sample size <50; one study lacked sufficient information to extract or calculate HRs and their CIs; and two studies were based on non-Asian populations. Finally, 10 studies were included in this meta-analysis (**Figure 1**; **Table 1**).

**Table 1:** Characteristics of all 10 studies included in the meta-analysis.

Author (year)	Country	Cancer type	N of cases	Duration	Age (years)	Gender (M/F)	Cutoff, mg/dl	Follow-up (months)	HR from	Outcome	NOS score
Tanaka (2015) (11)	Japan	UTUC	394	1995-2011	70 (IQR: 63-77)	289/105	390	30 (IQR:15-63)	UV/MV	OS CSS RFS	8
Huang (2017) (8)	China	UTUC	481	2002-2013	65.8 ± 11.1	311/170	422	40 (IQR:24-64)	UV/MV	OS CSS	8
Zhang (2016) (13)	China	UTUC	184	2006-2008	70 (61-75)	84/100	354	78 (34-92)	UV/MV	OS CSS	8
Liu (2019) (10)	China	UTUC	130	2009-2017	68 (IQR: 59.75-75)	90/40	360.2	30 (3-103)	UV/MV	CSS RFS PFS	7
Itami (2019) (9)	Japan	UTUC	125	1995-2016	72 (38-90)	96/29	340	51 (IQR:6-227)	UV/MV	OS RFS	7
Xu (2020) (12)	China	UTUC	703	2003-2016	67 (IQR: 59-74)	399/304	402.5	42 (1-168)	UV/MV	OS CSS RFS	8
Li (2019) (15)	China	BC	206	2012-2015	62 (19-83)	165/41	356	42 (5-72)	UV/MV	RFS PFS	7
Yang (2020) (17)	China	BC	145	2014-2019	65.92 ± 1016	125/20	314	NA	UV/MV	OS RFS	7
Gui (2021) (14)	China	BC	136	2005-2016	59.5 ± 6.7	101/35	339	NA	UV/MV	OS	7
Song (2022) (16)	China	BC	371	2013-2019	61.30 ± 12.82	291/80	370	NA	UV/MV	OS	7

UTUC, upper tract urothelial carcinoma; BC, Bladder cancer; IQR, interquartile range; MV, multivariate; UV, univariate; OS, overall survival; CSS, cancer-specific survival; RFS, recurrence-free survival; PFS, progression free survival.

The included studies had 2875 cases. 6 studies examined the prognostic value of plasma fibrinogen in UTUC (including 2017 patients), and 4 in BC (including 858 patients). As for survival outcomes, 8 studies (including 2539 patients) evaluated the prognostic value of plasma fibrinogen in predicting OS, 5 studies (including 1892 patients) evaluated CSS, 6 studies (including 1703 patients) evaluated RFS, and 2 studies (including 336 patients) evaluated PFS. All included studies achieved a minimum score of 7 on the NOS and were deemed to be of high quality.

### 3.2 Overall survival

**Figure 2:**
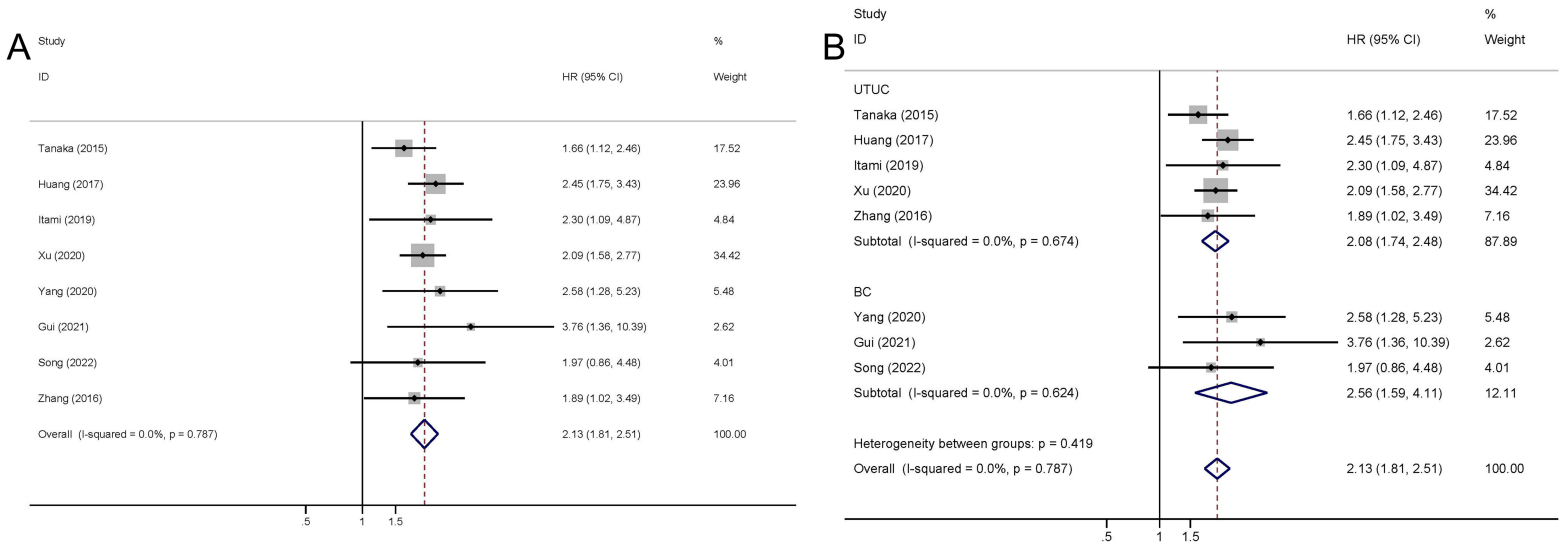
Meta-analysis of the association between the preoperative plasma fibrinogen and OS in urothelial cancers. Forest plot of studies evaluating pooled HR for OS in urothelial cancers **(A)** Forest plots of subgroup analyses by cancer type **(B)** for OS.

The presence of elevated preoperative plasma fibrinogen levels was found to be significantly associated with a poorer OS outcome in patients with UCs (fixed effect model, pooled hazard ratio: 2.13, 95% confidence interval: 1.81-2.51; P<0.001) (**Figure 2A**; **Table 2**). No heterogeneity across studies was found (I^2^ = 0.0%, P=0.787). Subgroup analysis based on cancer type revealed that high preoperative plasma fibrinogen was associated with poor OS in both UTUC (fixed effect model, pooled HR: 2.08, 95% CI: 1.74-2.48, P<0.001), and BC (fixed effect model, pooled HR: 2.56, 95% CI: 1.60-4.11, P<0.001) (**Figure 2B**; **Table 2**). In subgroup analyses based on country, high preoperative plasma fibrinogen was associated with poor OS in both Japan (fixed effect model, pooled HR: 1.78, 95% CI: 1.26-2.52, P=0.001), and China (fixed effect model, pooled HR: 2.24, 95% CI: 1.86-2.71, P<0.001) (**Table 2**). Additionally, statistically significant pooled HRs were also calculated in subgroup analysis when stratified by cutoff value (**Table 2**).

**Table 2:** HR values for OS according to subgroup analysis.

Categories	Study (cases)	Model	HR (95% CI)	Z	P_-_value	Heterogeneity	
						I^2^	P_H_-value
Overall	8 (2539)	Fixed	2.13 (1.81-2.51)	9.01	<0.001*	0.0%	0.787
Cancer type							
UTUC	5 (1887)	Fixed	2.08 (1.74-2.48)	8.16	<0.001*	0.0%	0.674
BC	3 (652)	Fixed	2.56 (1.60-4.11)	3.89	<0.001*	0.0%	0.624
Country							
Japan	2 (519)	Fixed	1.78 (1.26-2.52)	3.25	0.001*	0.0%	0.450
China	6 (2020)	Fixed	2.24 (1.86-2.71)	8.48	<0.001*	0.0%	0.842
Cut-off value; mg/dl							
<365	4 (590)	Fixed	2.36 (1.63-3.40)	4.58	<0.001*	0.0%	0.710
≥365	4 (1949)	Fixed	2.08 (1.73-2.50)	7.78	<0.001*	0.0%	0.534

OS, overall survival; HR, hazard ratio; CI, confidence interval; P_H_, P for heterogeneity; UTUC, upper tract urothelial carcinoma; BC, bladder cancer.

*P<0.05.

### 3.3 Recurrence-free survival

**Figure 3:**
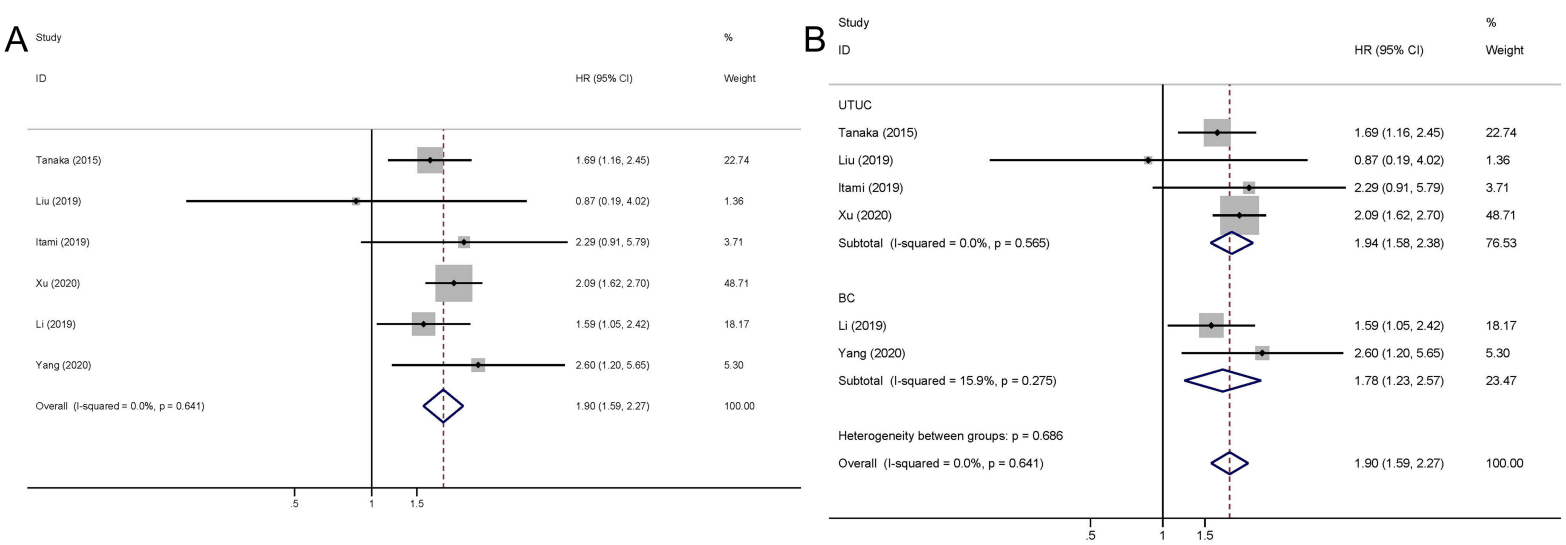
Meta-analysis of the association between the preoperative plasma fibrinogen and RFS in urothelial cancers. Forest plot of studies evaluating pooled HR for RFS in urothelial cancers **(A)** Forest plots of subgroup analyses by cancer type **(B)** for RFS.

The pooled outcome suggested that high preoperative plasma fibrinogen was significantly associated with short RFS in UCs (fixed effect model, pooled HR: 1.90, 95% CI: 1.59-2.27; P<0.001) with no heterogeneity across studies (I^2 =^ 0.0%, P=0.640) (**Figure 3A**; **Table 3**). Subgroup analysis based on cancer type revealed that high preoperative plasma fibrinogen was associated with poor RFS in both UTUC (fixed effect model, pooled HR: 1.94, 95% CI: 1.58-2.38, P<0.001), and BC (fixed effect model, pooled HR: 1.78, 95% CI: 1.23-2.57, P=0.002) (**Figure 3B**; **Table 3**). In subgroup analyses based on country, high preoperative plasma fibrinogen was associated with poor OS in both Japan (fixed effect model, pooled HR: 1.76, 95% CI: 1.28-2.49, P=0.001), and China (fixed effect model, pooled HR: 1.95, 95% CI: 1.59-2.41, P<0.001). Different cut-off value also showed prognostic value of preoperative plasma fibrinogen for RFS (**Table 3**).

**Table 3:** HR values for RFS according to subgroup analysis.

Categories	Study (cases)	Model	HR (95% CI)	Z	P_-_value	Heterogeneity	
						I^2^	P_H_-value
Overall	6 (1703)	Fixed	1.90 (1.59-2.27)	7.06	<0.001*	0.0%	0.640
Cancer type							
UTUC	4 (1352)	Fixed	1.94 (1.58-2.38)	6.38	<0.001*	0.0%	0.565
BC	2 (351)	Fixed	1.78 (1.23-2.57)	3.07	0.002*	15.9%	0.275
Country							
Japan	2 (519)	Fixed	1.76 (1.28-2.49)	3.21	0.001*	0.0%	0.551
China	4 (1184)	Fixed	1.95 (1.59-2.41)	6.31	<0.001*	0.0%	0.426
Cut-off value, mg/dl							
<365	4 (606)	Fixed	1.78 (1.27-2.48)	6.22	<0.001*	0.0%	0.358
≥365	4 (1097)	Fixed	1.95 (1.58-2.41)	3.38	0.001*	0.0%	0.509

RFS, recurrence-free survival; HR, hazard ratio; CI, confidence interval; P_H_, P for heterogeneity; UTUC, upper tract urothelial carcinoma; BC, bladder cancer.

*P<0.05.

### 3.4 Cancer-specific survival and Progression free survival

**Figure 4:**
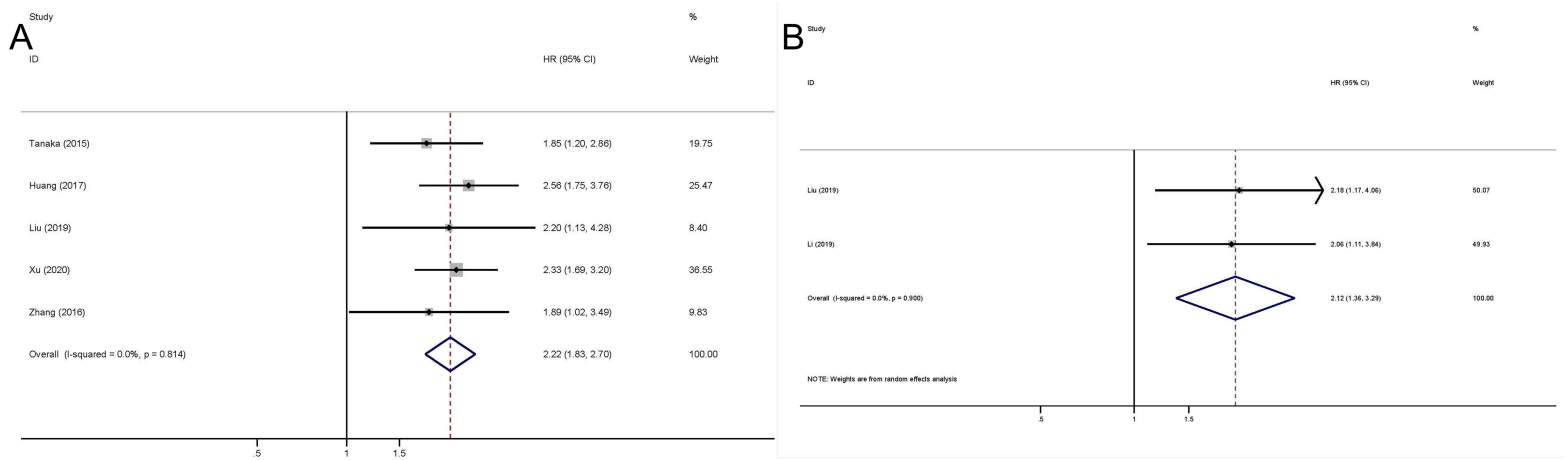
Meta-analysis of the association between the preoperative plasma fibrinogen and CSS/RFS in urothelial cancers. Forest plot of studies evaluating pooled HR for CSS **(A)** and for PFS **(B)**.

The pooled outcome suggested that the high preoperative plasma fibrinogen was significantly associated with short CSS among UC patients (fixed effect model, pooled HR: 2.22, 95% CI: 1.83-2.70; P<0.001) with no heterogeneity across studies (I^2^ = 0.0%, P=0.814) (**Figure 4**; **Table 4**). Additionally, high preoperative plasma fibrinogen was associated with poor PFS in UCs (fixed effect model, pooled HR: 2.12, 95% CI: 1.36-3.29, P=0.001). And no heterogeneity across studies was found (I^2 =^ 0.0%, P=0.900) (**Figure 4**; **Table 4**).

**Table 4:** HR values for CSS and PFS.

Categories	Study (cases)	Model	HR (95% CI)	Z	P_-_value	Heterogeneity	
						I^2^	P_H_-value
CSS	5 (1892)	Fixed	2.22 (1.83-2.70)	8.11	<0.001*	0.0%	0.814
PFS	2 (336)	Fixed	2.12 (1.36-3.29)	3.34	0.001*	0.0%	0.900

CSS, cancer-specific survival; PFS, progression free survival; HR, hazard ratio; CI, confidence interval; P_H_, P for heterogeneity.

*P<0.05.

### 3.5 Sensitivity analysis

The results of sensitivity analysis for OS, CSS, and RFS outcomes demonstrated that the conclusions for OS, CSS, and RFS remained stable because the pooled HRs were not significantly influenced by excluding any individual study (**Supplementary Figure 1**).

### 3.6 Publication bias

**Figure 5:**
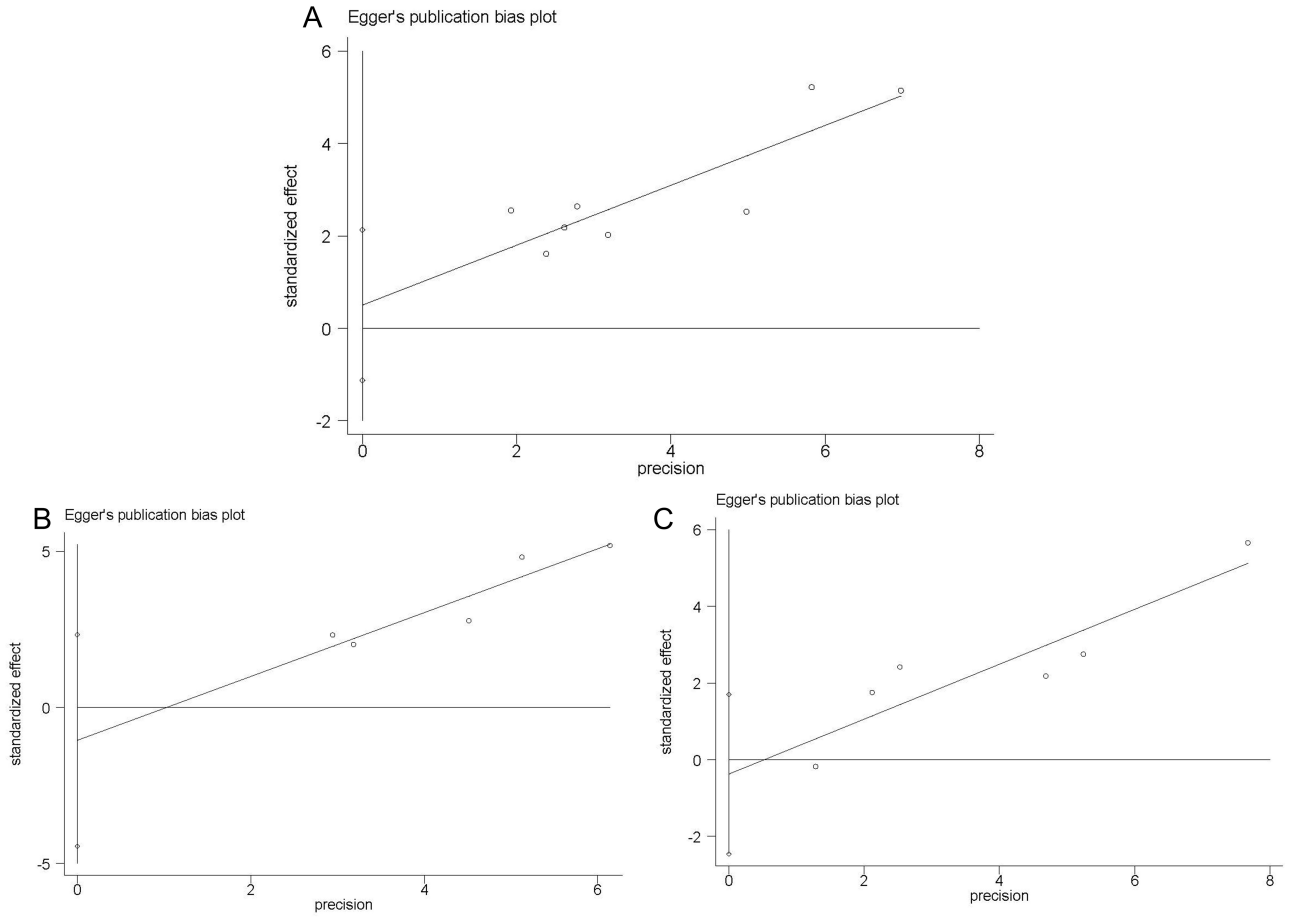
Egger’s linear regression test. Egger’s publication bias plots for OS **(A)** CSS **(B)** and RFS **(C)**.

The presence of publication bias in the included investigations was assessed using Begg’s test and Egger’s linear regression test. In Begg’s test, we found that P-value of 0.536 for OS, 0.806 for CSS, and 0.260 for RFS. In Egger’s test, the corresponding P-values were found to be 0.480 for OS (**Figure 5A**), 0.394 for CSS (**Figure 5B**), and 0.639 for RFS (**Figure 5C**). Thus, our meta-analysis did not reveal any significant publication bias.

## 4 Discussion

Our meta-analysis incorporated a total of 2875 cases from 10 eligible studies, which were deemed of high quality based on the NOS score system. The results of our study suggest that preoperative plasma fibrinogen levels can serve as a reliable predictor for oncologic outcomes in patients with localized UC. Elevated preoperative plasma fibrinogen levels are associated with unfavorable OS, CSS, RFS, and PFS in patients with UTUC or BC. In subgroup analyses, BC shown a better predictive value for OS, suggesting that preoperative plasma fibrinogen has the best predictive value for OS in BC. UTUC demonstrated a better predictive value for RFS, indicating that preoperative plasma fibrinogen has the best predictive value for RFS in UTUC. Additionally, we found that the preoperative plasma fibrinogen has a better predictive value for OS and RFS among the Chinese population compared to the Japanese population. Therefore, preoperative plasma fibrinogen could serve as a cost-effective and readily accessible prognostic biomarker for urothelial cancers in the Asian population, despite variations in effect sizes.

Although UTUC and BC share some common risk factors, however, they exhibit distinct biological, practical, and clinical characteristics (20), which may account for the difference in prognostic value of preoperative plasma fibrinogen between these two types of UC. The subgroup analysis based on country in our meta-analysis revealed that preoperative plasma fibrinogen exhibited a stronger predictive value within the Chinese population compared to the Japanese population, potentially attributed to limited study availability and inadequate sample sizes. Further studies should be conducted to validate these insignificant results. Sensitivity analyses confirmed the stableness of the pooled outcomes.

Negative associations between preoperative plasma fibrinogen and oncological prognosis have been reported in numerous cancers, not limited to urothelial cancers. These included renal cell carcinoma (21), prostate cancer (22), gastric cancer (23), laryngeal squamous cell carcinoma (24), lung cancer (25), hepatocellular carcinoma (26), and pancreatic cancer (27). However, the underlying mechanisms for the associations have not been clearly elucidated. Previous *in vitro* studies have verified that fibrinogen can promote cancer cell proliferation, invasion, epithelial-to-mesenchymal transition (EMT), angiogenesis, and hematogenous dissemination transition (28, 29). Thus, fibrinogen could play an important role in tumor progression. The previous studies have demonstrated the ability of fibrinogen to interact with secreted growth factors, such as transforming growth factor-β (TGF-β), fibroblast growth factor-2 (FGF-2), vascular growth factor (VEGF), and platelet-derived growth factor (PDGF) to stimulate tumor cell proliferation and angiogenesis (30–32). In esophageal squamous cell carcinoma, Zhang et al. (33) have demonstrated that fibrinogen can promote malignant biological tumor behavior involving EMT via the p-AKT/p-mTOR pathway. However, the exact biological mechanism for the relationship between elevated plasma fibrinogen and poor prognosis of UC remains unknow. Further investigations are needed to explore the underlying mechanism.

Despite advancements in the management of cancer, some patients still face a poor prognosis due to local tumor recurrence or distant metastasis. Therefore, novel biomarkers are necessary to predict the prognoses accurately and formulate follow-up strategies based on the stratification of risks for UC patients. Tumor-related immune responses in tumor micro-environment serve as immunological surveillance and contribute to antitumor immune responses, which are closely associated with patients’ tumor outcomes (34). Therefore, certain immune-inflammatory indicators, such as C-reactive protein (CRP) (35), platelet-lymphocyte ratio (PLR) (36), neutrophil-lymphocyte ratio (NLR) (37), lymphocyte-monocyte ratio (LMR) (38), albumin (39), and plasma fibrinogen levels (40), have been reported as potential biomarkers for diagnosing and predicting the prognosis of tumor patients. The findings of this meta-analysis indicate that plasma fibrinogen serves as a valuable prognostic biomarker, enabling the identification of high-risk UC patients prior to treatment and subsequently enhancing their tumor outcomes. Esumi et al. (41) also reported that inhibiting coagulation events by using r-hirudin, a highly specific thrombin inhibitor, significantly inhibited lung metastasis in an animal model. Thus, the administration of anticoagulants may potentially mitigate hematogenous metastasis in UC patients exhibiting elevated levels of plasma fibrinogen.

Although our study comprehensively assessed the prognostic value of the preoperative plasma fibrinogen in UCs with no obvious heterogeneity and publication bias, it had certain limitations. Firstly, some of the included studies only enrolled a small number of patients, which might introduce confounder bias. However, excluding these studies did not significantly affect the overall estimation. Secondly, our focus was primarily on the post-surgical outcomes, thereby excluding consideration of other treatment modalities. Consequently, this led to a paucity of data within the studies included. Additionally, our meta-analysis included a limited number of studies. However, our meta-analysis exhibited no significant heterogeneity and publication bias. Besides, sensitivity analysis confirmed that our findings were stable and reliable. Finally, all the studies included in this meta-analysis were retrospective observational studies with inherent structural defects; therefore, we cannot draw definitive conclusions regarding how preoperative plasma fibrinogen influences oncologic outcomes.

In conclusion, the findings of our meta-analysis indicate a significant association between elevated preoperative plasma fibrinogen levels and unfavorable tumor outcomes in UCs. While further studies are needed, our findings suggested that elevated preoperative plasma fibrinogen could serve as a potential prognostic biomarker for UC patients and may influence clinical decision-making.

## Data Availability

The original contributions presented in the study are included in the article/**Supplementary Material**. Further inquiries can be directed to the corresponding author/s.

## Supplementary material

Supplementary Figure 1 Sensitivity analysis for OS **(A)** CSS **(B)** and RFS **(C)**.

## References

[1] Rouprêt M Seisen T Birtle AJ Capoun O Compérat EM Dominguez-Escrig JL . European association of urology guidelines on upper urinary tract urothelial carcinoma: 2023 update. Eur Urol. (2023) 84:49–64. doi: 10.1016/j.eururo.2023.03.013 36967359

[2] Siegel RL Miller KD Wagle NS Jemal A . Cancer statistics, 2023. CA Cancer J Clin. (2023) 73:17–48. doi: 10.3322/caac.21763 36633525

[3] He W Chen C Lin T Xu Q Ye C Du J . Epidemiology, treatments, and related biomarkers of locally advanced or metastatic urothelial carcinoma in Chinese population: A scoping review. Cancer Med. (2023) 12:15384–403. doi: 10.1002/cam4.6112 PMC10417093 37387501

[4] Neerman-Arbez M Casini A . Fifty years of fibrinogen structure and function. Semin Thromb Hemost. (2024) 50:148–50. doi: 10.1055/s-0043-1775857 37813370

[5] Mohammadinejad A Aleyaghoob G Nooranian S Dima L Moga MA Badea M . Development of biosensors for detection of fibrinogen: a review. Anal Bioanal Chem. (2024) 416:21–36. doi: 10.1007/s00216-023-04976-1 37837539

[6] Tinholt M Sandset PM Iversen N . Polymorphisms of the coagulation system and risk of cancer. Thromb Res. (2016) 140 Suppl 1:S49–54. doi: 10.1016/S0049-3848(16)30098-6 27067978

[7] Sharma BK Flick MJ Palumbo JS . Cancer-associated thrombosis: A two-way street. Semin Thromb Hemost. (2019) 45:559–68. doi: 10.1055/s-0039-1693472 31382306

[8] Huang J Yuan Y Wang Y Zhang J Kong W Chen H . Prognostic value of preoperative plasma fibrinogen level and platelet-to-lymphocyte ratio (F-PLR) in patients with localized upper tract urothelial carcinoma. Oncotarget. (2017) 8:36761–71. doi: 10.18632/oncotarget.13611 PMC5482695 27901490

[9] Itami Y Miyake M Tatsumi Y Gotoh D Hori S Morizawa Y . Preoperative predictive factors focused on inflammation-, nutrition-, and muscle-status in patients with upper urinary tract urothelial carcinoma undergoing nephroureterectomy. Int J Clin Oncol. (2019) 24:533–45. doi: 10.1007/s10147-018-01381-y 30604161

[10] Liu R Zhou X Zou L Chen Q Hu Y Hu J . Clinicopathological and prognostic significance of preoperative plasma fibrinogen level in patients with upper urinary tract urothelial carcinoma: A retrospective tumor marker prognostic study. Int J Surg. (2019) 65:88–93. doi: 10.1016/j.ijsu.2019.03.022 30951871

[11] Tanaka N Kikuchi E Kanao K Matsumoto K Shirotake S Miyazaki Y . Impact of combined use of blood-based inflammatory markers on patients with upper tract urothelial carcinoma following radical nephroureterectomy: proposal of a cumulative marker score as a novel predictive tool for prognosis. Eur Urol Focus. (2015) 1:54–63. doi: 10.1016/j.euf.2015.02.001 28723357

[12] Xu H Ai JZ Tan P Lin TH Jin X Gong LN . Pretreatment elevated fibrinogen level predicts worse oncologic outcomes in upper tract urothelial carcinoma. Asian J Androl. (2020) 22:177–83. doi: 10.4103/aja.aja_38_19 PMC7155795 31169138

[13] Zhang B Song Y Jin J Zhou LQ He ZS Shen C . Preoperative plasma fibrinogen level represents an independent prognostic factor in a chinese cohort of patients with upper tract urothelial carcinoma. PloS One. (2016) 11:e0150193. doi: 10.1371/journal.pone.0150193 26930207 PMC4773108

[14] Gui H Song Y Yin Y Wang H Rodriguez R Wang Z . Prognostic value of preoperative inflammation-based predictors in patients with bladder carcinoma after radical cystectomy. Open Med (Wars). (2021) 16:816–25. doi: 10.1515/med-2021-0277 PMC8142381 34056114

[15] Li X Shu K Zhou J Yu Q Cui S Liu J . Preoperative plasma fibrinogen and D-dimer as prognostic biomarkers for non-muscle-invasive bladder cancer. Clin Genitourin Cancer. (2020) 18:11–19.e11. doi: 10.1016/j.clgc.2019.10.025 31787543

[16] Song Y Tian J Yang L Zhang Y Dong Z Ding H . Prognostic value of preoperative platelet-related parameters and plasma fibrinogen in patients with non-muscle invasive bladder cancer after transurethral resection of bladder tumor. Future Oncol. (2022) 18:2933–42. doi: 10.2217/fon-2022-0223 35880441

[17] Yang S Guan H Wang S Wu H Sun W Chen Z . Plasma fibrinogen predicts the prognosis of bladder cancer patients after radical cystectomy. Cancer manag Res. (2020) 12:9303–14. doi: 10.2147/CMAR.S269244 PMC7532920 33061620

[18] Song H Kuang G Zhang Z Ma B Jin J Zhang Q . The prognostic value of pretreatment plasma fibrinogen in urological cancers: A systematic review and meta-analysis. J Cancer. (2019) 10:479–87. doi: 10.7150/jca.26989 PMC6360290 30719143

[19] Jiang C Perimbeti S Deng L Xing J Chatta GS Han X . Medicaid expansion and racial disparity in timely multidisciplinary treatment in muscle invasive bladder cancer. J Natl Cancer Inst. (2023) 115:1188–93. doi: 10.1093/jnci/djad112 37314971

[20] Soria F Shariat SF Lerner SP Fritsche HM Rink M Kassouf W . Epidemiology, diagnosis, preoperative evaluation and prognostic assessment of upper-tract urothelial carcinoma (UTUC). World J Urol. (2017) 35:379–87. doi: 10.1007/s00345-016-1928-x 27604375

[21] Ni J Wang Y Zhang H Wang K Song W Luo M . Combination of preoperative plasma fibrinogen and neutrophil-to-lymphocyte ratio to predict the prognosis for patients undergoing laparoscopic nephrectomy for renal cell carcinoma. Am J Cancer Res. (2022) 12:3713–28. PMC9442019 36119818

[22] Man YN Chen YF . Systemic immune-inflammation index, serum albumin, and fibrinogen impact prognosis in castration-resistant prostate cancer patients treated with first-line docetaxel. Int Urol Nephrol. (2019) 51:2189–99. doi: 10.1007/s11255-019-02265-4 31456101

[23] Zhang Y Liu N Liu C Cao B Zhou P Yang B . High fibrinogen and platelets correlate with poor survival in gastric cancer patients. Ann Clin Lab Sci. (2020) 50:457–62. 32826241

[24] Cai H Zhang ZH Zhou YJ Liu J Chen HQ Lin RY . The prognostic value of preoperative plasma fibrinogen and neutrophil-to-lymphocyte ratio in patients with laryngeal squamous cell carcinoma. Ear Nose Throat J. (2021) 100:731–6. doi: 10.1177/0145561320920746 32380854

[25] Zhang K Xu Y Tan S Wang X Du M Liu L . The association between plasma fibrinogen levels and lung cancer: a meta-analysis. J Thorac Dis. (2019) 11:4492–500. doi: 10.21037/jtd.2019.11.13 PMC6940208 31903237

[26] Dai T Peng L Lin G Li Y Yao J Deng Y . Preoperative elevated plasma fibrinogen level predicts tumor recurrence and poor prognosis in patients with hepatocellular carcinoma. J Gastrointest Oncol. (2019) 10:1049–63. doi: 10.21037/jgo.2019.09.11 PMC6955016 31949922

[27] Chung KH Lee JC Lee J Cho IK Kim J Jang W . Serum fibrinogen as a diagnostic and prognostic biomarker for pancreatic ductal adenocarcinoma. Pancreatology. (2020) 20:1465–71. doi: 10.1016/j.pan.2020.06.010 32873483

[28] Staton CA Brown N J Lewis CE . The role of fibrinogen and related fragments in tumour angiogenesis and metastasis. Expert Opin Biol Ther. (2003) 3:1105–20. doi: 10.1517/14712598.3.7.1105 14519075

[29] Shu YJ Weng H Bao RF Wu XS Ding Q Cao Y . Clinical and prognostic significance of preoperative plasma hyperfibrinogenemia in gallbladder cancer patients following surgical resection: a retrospective and *in vitro* study. BMC Cancer. (2014) 14:566. doi: 10.1186/1471-2407-14-566 25096189 PMC4131047

[30] Sahni A Simpson-Haidaris PJ Sahni SK Vaday GG Francis CW . Fibrinogen synthesized by cancer cells augments the proliferative effect of fibroblast growth factor-2 (FGF-2). J Thromb Haemost. (2008) 6:176–83. doi: 10.1111/j.1538-7836.2007.02808.x 17949478

[31] Sahni A Odrljin T Francis CW . Binding of basic fibroblast growth factor to fibrinogen and fibrin. J Biol Chem. (1998) 273:7554–9. doi: 10.1074/jbc.273.13.7554 9516457

[32] Sahni A Francis CW . Vascular endothelial growth factor binds to fibrinogen and fibrin and stimulates endothelial cell proliferation. Blood. (2000) 96:3772–8. doi: 10.1182/blood.V96.12.3772 11090059

[33] Zhang F Wang Y Sun P Wang ZQ Wang DS Zhang DS . Fibrinogen promotes Malignant biological tumor behavior involving epithelial-mesenchymal transition via the p-AKT/p-mTOR pathway in esophageal squamous cell carcinoma. J Cancer Res Clin Oncol. (2017) 143:2413–24. doi: 10.1007/s00432-017-2493-4 PMC11819069 28801734

[34] Sfanos KS Yegnasubramanian S Nelson WG De Marzo AM . The inflammatory microenvironment and microbiome in prostate cancer development. Nat Rev Urol. (2018) 15:11–24. doi: 10.1038/nrurol.2017.167 29089606

[35] Sambataro D Politi MR Messina A Scarpello L Messina S Guggino R . Relationship of inflammatory parameters and nutritional status in cancer patients. Anticancer Res. (2023) 43:2821–9. doi: 10.21873/anticanres.16451 37247899

[36] Zhou H Li J Zhang Y Chen Z Chen Y Ye S . Platelet-lymphocyte ratio is a prognostic marker in small cell lung cancer-A systemic review and meta-analysis. Front Oncol. (2022) 12:1086742. doi: 10.3389/fonc.2022.1086742 36713502 PMC9880219

[37] Wang H Gong H Tang A Cui Y . Neutrophil/lymphocyte ratio predicts lymph node metastasis in patients with gastric cancer. Am J Transl Res. (2023) 15:1412–20. PMC10006780 36915778

[38] Mei P Feng W Zhan Y Guo X . Prognostic value of lymphocyte-to-monocyte ratio in gastric cancer patients treated with immune checkpoint inhibitors: a systematic review and meta-analysis. Front Immunol. (2023) 14:1321584. doi: 10.3389/fimmu.2023.1321584 38090560 PMC10711042

[39] Xu H Zheng X Ai J Yang L . Hemoglobin, albumin, lymphocyte, and platelet (HALP) score and cancer prognosis: A systematic review and meta-analysis of 13,110 patients. Int Immunopharmacol. (2023) 114:109496. doi: 10.1016/j.intimp.2022.109496 36462339

[40] Bu F Cao S Deng X Zhang Z Feng X . Evaluation of C-reactive protein and fibrinogen in comparison to CEA and CA72-4 as diagnostic biomarkers for colorectal cancer. Heliyon. (2023) 9:e16092. doi: 10.1016/j.heliyon.2023.e16092 37215813 PMC10196578

[41] Esumi N Fan D Fidler IJ . Inhibition of murine melanoma experimental metastasis by recombinant desulfatohirudin, a highly specific thrombin inhibitor. Cancer Res. (1991) 51:4549–56. 1873799

