# Supplementary figures and images for: The prognostic value of preoperative plasma fibrinogen in Asian patients with urothelial cancer: a systematic review and meta-analysis
Source: Front Endocrinol (Lausanne). 2024 Aug 29;15:1360595. doi: 10.3389/fendo.2024.1360595 (PMC11390423; doi:10.3389/fendo.2024.1360595)

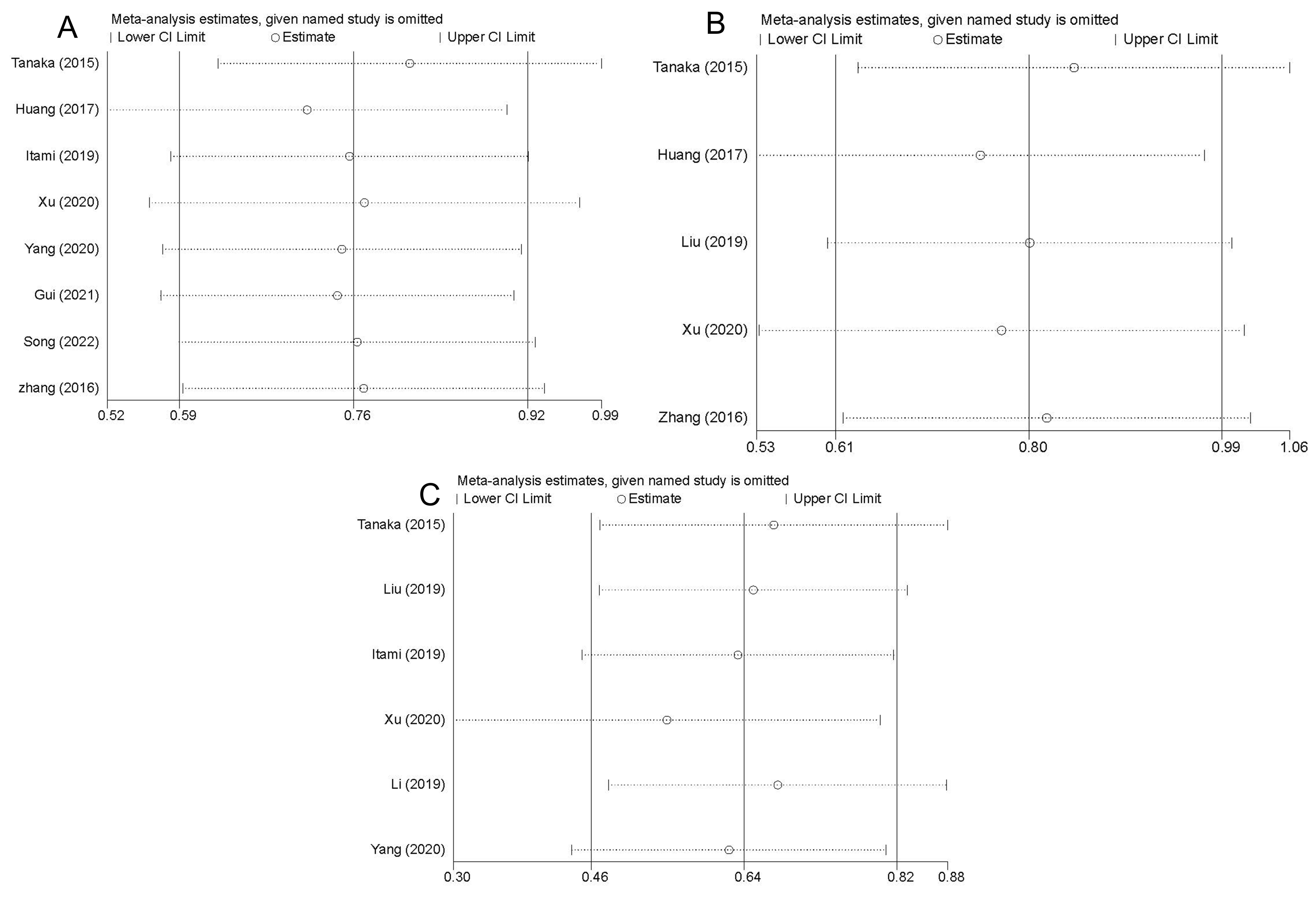

Supplement: Supplementary Figure 1 — Sensitivity analysis for OS (A) CSS (B) and RFS (C). [file Image1.jpeg]
